# Investigating the quality of Iranian hospitals’ websites and their association to the Province’s share of medical tourism

**DOI:** 10.1186/s13104-023-06619-1

**Published:** 2023-11-14

**Authors:** Fatemeh Shaygani, Soodeh Jahangiri, Mohammad Hassan Zahed Roozegar, Zahra Kavosi, Milad Ahmadi Marzaleh

**Affiliations:** 1 Student Research Committee, Shiraz University of Medical Sciences, Shiraz, Iran; 2 Health Tourism Student Scientific Association, Shiraz University of Medical Sciences, Shiraz, Iran; 3 Endocrine and metabolism research institute, University of medical sciences, Tehran, Iran; 4 Health Policy Research Center, Institute of Health, Shiraz University of Medical Sciences, Shiraz, Iran; 5 Health Human Resources Research Center, School of Health Management and Information Sciences, Shiraz University of Medical Sciences, Shiraz, Iran; 6 Department of Health in Disasters and Emergencies, Health Human Resources Research Center, School of Health Management and Information Sciences, Shiraz University of Medical Sciences, Shiraz, Iran

**Keywords:** Website, Hospital, Medical tourism, Health tourism, Iran

## Abstract

**Background:**

Nowadays, virtual methods are among the most important and influential marketing instruments in various industries, such as medical tourism. This study aims to investigate the quality of Iranian hospitals’ web pages and their association with the province’s share of the medical tourism industry in Iran and the ownership type of hospitals.

**Methods:**

In this analytical cross-sectional study, the quality of hospitals’ websites was investigated through a 36-item self-administered questionnaire which was validated, and its reliability was verified (Cronbach’s alpha = 74%.). The questionnaire was categorized into three sections: hospital services and facilities, hospital’s medical tourism-related services, and tourism information of the destination province. The census method was used for data collection. Data analysis was performed using the independent t-test and analysis of variance in SPSS software (version 25), and a P-value < 0.05 was considered statistically significant.

**Results:**

A total of, 102 hospitals with an IPD (International Patients Department) were included in the study, and 21.6% did not have an English-language page and were excluded from the study. The mean total score was 47 ± 7.5, indicating low-quality content. Public hospitals had lower quality scores than semi-private and private hospitals. The total quality score, information about the hospital and its services, and the score of information about medical tourism-related services were associated with the province’s share of national medical tourism.

**Conclusion:**

According to the obtained results and the possible role of website quality in increasing provinces’ medical tourism development, the IPD page on hospital websites should be revised and regularly updated to make them more informative for prospective medical tourists.

## Introduction

According to the World Tourism Organization (UNWTO), “health tourism” is organized travel that primarily promotes physical, mental, and/or spiritual well-being, using medical or wellness-based activities [1]. Health tourism occurs for several reasons in the origin country, such as the high price of healthcare services [2–5], long waiting lists [2, 4], dissatisfaction with the healthcare services [6], lack of a specific medical service or technology, and legal prohibition of specific medical services [2, 3]. This industry is considered one of the most important means of revenue generation and job creation in healthcare [7]. In 2020, the industry’s revenue was approximately $55 billion globally and was expected to grow remarkably, reaching over $200 billion by 2027 [8].

Iran is considered one of the leading countries in the field of health tourism in the Middle East region attracting a lot of health tourists annually, especially from the Arab world [9]. Iran has several wonderful natural, religious, historical, and cultural tourist attractions as well as many modern hospitals and medical centers with up-to-date equipment [9]. It is a popular health tourism destination as it provides low-cost treatments and healthcare services, low waiting times for treatments, internationally famous physicians and credible surgeons, and successful results of surgical interventions at the global level [10].

The importance of virtual marketing and health tourism websites is greatly emphasized in the literature, indicating their role in the development of this industry [11–13]. A standard and multilingual website providing necessary information for foreign patients makes medical hospitals and centers more tourist-friendly and efficient [13–15]. Receiving comprehensive, transparent information for patients abroad, especially those planning to take a medical trip for the first time can play a decisive role in choosing medical centers and even the destination country [14]. Therefore, to find out whether the quality of Iranian hospital websites has been effective in the development of health tourism in that province in Iran, after assessing their quality, the association between the quality of hospital websites and provinces’ share of the medical tourism industry was investigated. Also, the association of the quality of Iranian hospital websites to the ownership type of hospitals was explored.

## Methods

### Data collection

The census method was used for data collection in this analytical cross-sectional study, which was carried out from December 2022 to February 2023. Three independent researchers reviewed the websites of all hospitals in the province capitals according to the inclusion criteria: (1) Those that had an international patient department (IPD) and (2) are located in provinces with high share (≥ 1%) / low share (< 1%) of the national medical tourism industry based on the study of Amiri et al. [16]. The province capitals with high share (72 IPDs) included: Tehran, Khorasan Razavi, Qom, Fars, Khuzestan, West Azerbaijan, East Azerbaijan, Hormozgan, Semnan, and Kermanshah. The province capitals that had a low share (30 IPDs), included South Khorasan, North Khorasan, Isfahan, Alborz, Lorestan, Kerman, Hamedan, Golestan, Gilan, and Mazandaran. Hospitals without an English-language website (n = 22) were excluded from the study.

The data collection instrument in this study was a self-administered questionnaire consisting of 36 items in three sections: hospital services and facilities (15 questions), information about the hospital’s medical tourism services (13 questions), and guide information for tourism in the province (8 questions). The questionnaire was designed and scored on a 5-point Likert scale (complete and up-to-date information = 4, complete and old information = 3, incomplete and up-to-date information = 2, incomplete and old information = 1, lack of information = 0). The total score for each hospital was calculated as the sum of the scores of all questions, with a higher score indicating a higher level of quality. The minimum and maximum scores were between 0 and 144. Moreover, we divided the quality of hospitals’ websites into “low,” “average,“ and “high” if they achieved less than 40%, 40–70%, and more than 70% of the total achievable score, respectively.

An expert panel approved the face and content validity of the questionnaire from professors and researchers in health services management and health information management. To check the reliability, two hospitals with English websites were randomly selected, and three researchers scored them separately; Cronbach’s alpha was calculated at 74%.

### Data analysis

This study analyzed data using IBM SPSS Statistics software (version 25). Quality assurance was performed by supervision during data collection, extraction, entry into the software, and analysis. Data analysis was performed using the independent t-test and analysis of variance. A P-value of less than 0.05 was considered statistically significant in the analysis.

### Ethical consideration

The proposal for this study was approved by the Ethics Committee affiliated with Shiraz University of Medical Sciences (SUMS), Shiraz, Iran, and encoded as IR.SUMS.NUMIMG.REC.1402.013. In addition, the confidentiality of the data was taken very seriously and all methods were performed following the relevant guidelines and regulations (Declaration of Helsinki). Informed consent forms do not apply to this study and have not been used.

## Results

Hospital IPDs in the cities of Tehran, Mashhad, Qom, Shiraz, Ahvaz, Urmia, Tabriz, Bandar Abbas, Semnan, Kermanshah, Birjand, Bojnord, Isfahan, Karaj, Khorramabad, Kerman, Hamedan, Gorgan, Rasht, and Sari were selected )102 hospitals). Twenty-two (21.6%) hospitals did not have English websites and were excluded from the study; 80 IPDs were included. The private provider-to-public provider ratio was 1.5, and the number of semi-private IPDs was negligible (Table 1). The public, semi-private, and private IPDs obtained 25.5%, 26%, and 28% of the total achievable score, respectively. Only 4 IPDs achieved between 40% and 70%, considered an average score, and the rest obtained a low score (lower than 40%).

**Table 1:** Features of included IPDs

Ownership type of the hospital	Provinces of hospitals	
	**A high share of medical tourism n (%)**	**A low share of medical tourism n (%)**
Public	20(25)	11(13.7)
Semi-private	1(1.2)	1(1.2)
Private	36(45)	11(13.7)
Total	57(71.2%)	23(28.7%)

**Fig. 1:**
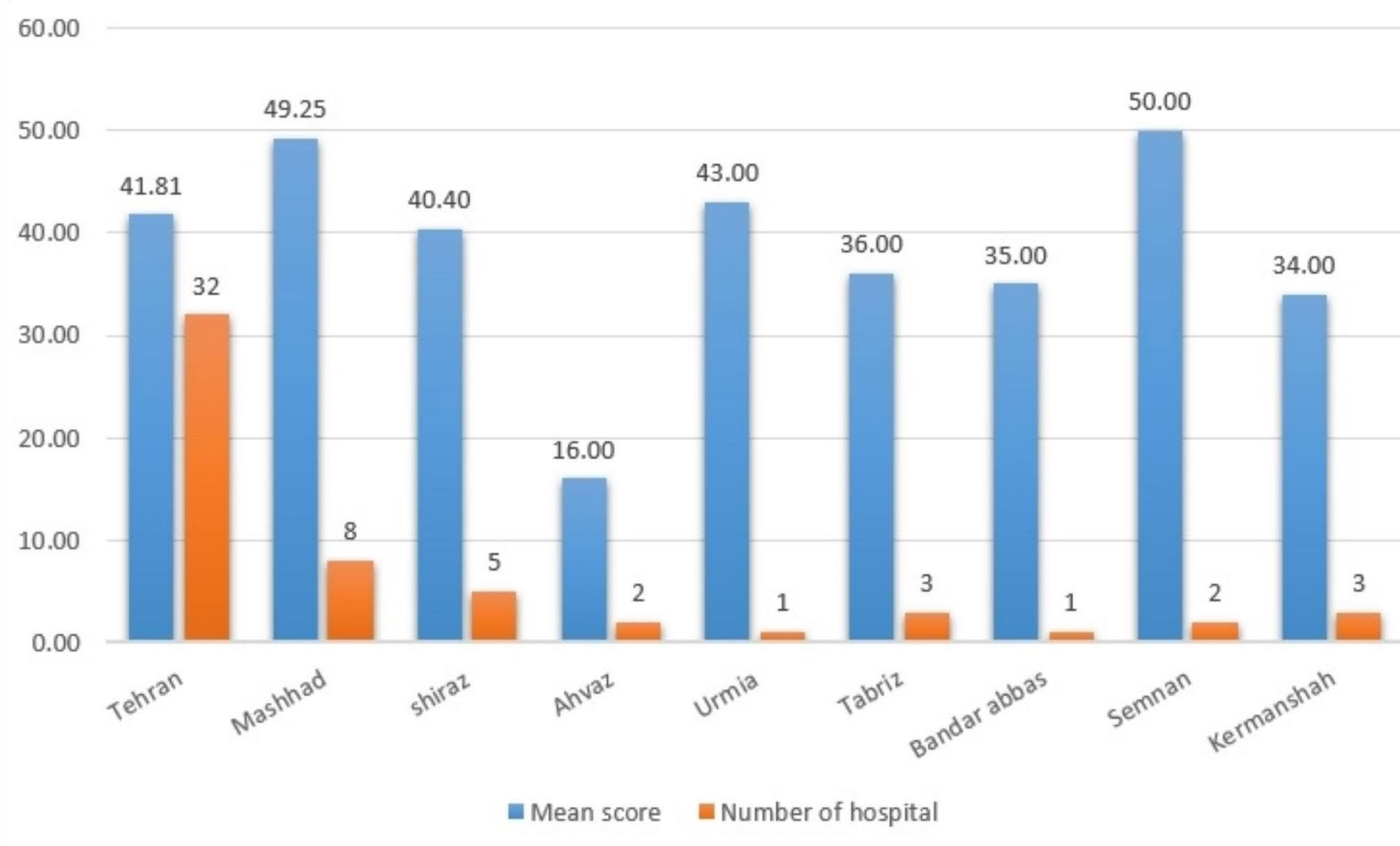
Characteristics of IPDs in provinces with a high share of medical tourism

**Fig. 2:**
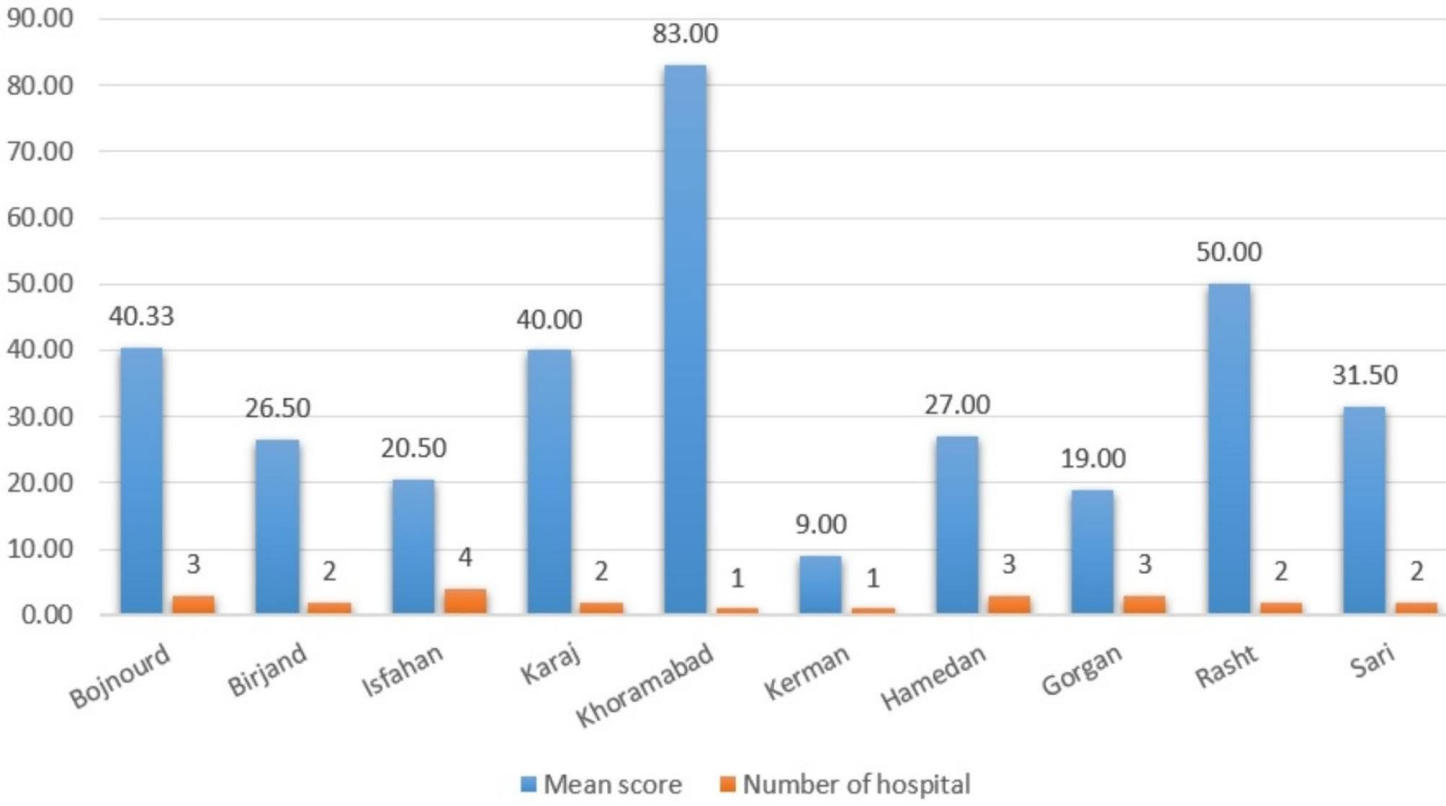
Characteristics of IPDs in provinces with a low share of medical tourism

According to the results, the mean quality score of all included IPDs’ websites was 47 ± 7.5, demonstrating low-quality content. Figures 1 and 2 give an understanding of the quality score of IPDs’ websites in each province’s capital. It is possible to compare scores with other provinces considering the number of included IPDs in each province.

According to Fig. 1, Semnan, Mashhad, and Urmia achieved the highest mean scores among cities with a high share of national medical tourism. The IPDs of Qom were not included in this figure because the IPDs of this city did not have English websites, so they were excluded.

Also, the IPDs of Khorramabad, Rasht, and Bojnord were top-ranked among those with a low share of national medical tourism (Fig. 2).

According to Table 2, the mean score of the first two sections was higher in provinces with a high share of medical tourism. The quality of information was very low regarding the third part of the questionnaire and was not different in provinces with high and low shares of medical tourism. Moreover, the mean total score of website quality was about 1.3 times higher for hospitals with a high share of medical tourism than those with a low share. Univariate analysis showed that the mean score of the first section of the questionnaire (hospital services and facilities), the second section (hospital’s medical tourism services), and the total mean score were significantly associated with the province’s share of national medical tourism (P value < 0.05) (Table 2).

**Table 2:** The association between questionnaire scores in each section and hospital provinces

Score	Provinces of hospitals		P-value^¶^
	**Low share**	**High share**	
1st section (hospital services and facilities)	18.17 ± 10.31	23.64 ± 7.82	**0.012**
2nd section (hospital’s medical tourism services)	11.08 ± 6.17	15.98 ± 7.65	**0.008**
3rd section (tourism in the province)	2.43 ± 3.31	1.6 ± 2.94	0.318
Total	31.69 ± 17.71	41.29 ± 13.69	**0.011**

Data are presented as mean ± SD

^¶^ Independent T-test or Mann-Whitney U test

Significant values (p-value < 0.05) are in bold.

According to Table 3, the website quality mean score of each section of the questionnaire was not significant based on the ownership type of the hospital (Table 3).

**Table 3:** The association between questionnaire scores in each section and ownership of the hospitals

Score	Ownership type of the hospitals			P-value^¶^	
	**Public**	**Semi-private**	**Private**		
1st section (hospital services and facilities)	20.25 ± 6.17	20.5 ± 9.19	23.34 ± 10.27	0.320	
2nd section (hospital’s medical tourism services)	14.7 ± 8.39	15.5 ± 9.19	14.44 ± 7.08	0.974	
3rd section (tourism in the province)	1.7 ± 2.52	1.5 ± 2.12	1.97 ± 3.42	0.857	
Total	36.74 ± 13.6	37.5 ± 16.26	39.76 ± 16.8	0.702	

Data are presented as mean ± SD

^¶^ Analysis of variance or Kruskal-Wallis tests

## Discussion

These days, virtual marketing is one of the most important and effective elements of product promotion [17, 18]. Studies show that having a standard website with appropriate information for the users effectively increases the probability of their willingness to receive services [11, 13, 19]. Medical tourism, as one of the most important and profitable health-related industries, is not an exception; providing adequate information for foreign patients on the websites of hospitals and healthcare centers can help to attract them and turn them into loyal customers [20, 21].

According to the findings of this study, an average of Iranian IPDs’ websites had low-quality content. Similarly, a study by Qolipour et al., showed that there was a very high gap between medical tourists’ perception and expectations regarding the hospital’s website providing adequate information and the websites could not meet the medical tourists’ needs [22]. Likewise, a study by Shaarbafchi Zadeh demonstrated that the websites of IPDs in Isfahan City, Iran were not satisfactory [23]. Also, our study reported that all ownership types of the hospitals (public, semi-private, and private) had low-quality content. On the other side, Abdekhoda et al.‘s research showed that the websites of public hospitals of Tehran University of Medical Sciences scored 50% regarding content criteria, which was considered an average quality [24]. Also in contrast to our study, research on private hospitals in three competing Asian countries (India, Malaysia, and Thailand) revealed that medical tourism websites’ qualities were relatively strong, moderate, and moderate, respectively [13]. This further highlights the need to improve Iranian hospitals’ website quality to promote health tourism in the country.

Moreover, according to our findings, while all ownership types of the hospitals had low-quality content, public hospitals had lower quality scores than semi-private and private hospitals. Similarly, a study by Baghbanian et al. showed that among the medical tourism websites, the websites of private organizations scored better [25]. This may be because private sector hospitals do not receive a governmental budget and are fully responsible for their financing, so they attract more foreign patients through their IPD websites than public sector hospitals. Therefore, while the quality of the websites of all hospitals must be improved regardless of their ownership type, a more fundamental change should be considered for public hospitals in this respect.

Furthermore, based on our results, the IPDs’ website information regarding hospital services and facilities and the hospitals’ medical tourism-related services was reported to be very important and played a significant role in increasing the province’s development in the medical tourism industry. Likewise, a study by Lee et al. demonstrated that the most important persuasive factor for medical tourists on the hospitals’ websites was adequate and appropriate information about hospitals’ medical services and claimed that websites and social media play a key role in attracting medical tourists and in the industry development [14].

In addition, our research shows that guide information for tourism in the province was not found to be a significant factor in developing the industry, however, a study by Samadbeik et al. [26] mentioned that tourism information is an effective factor in the richness of medical tourism websites’ content and indicated that this information can provide medical tourists with all information they may need. Therefore, even though it was not proven that this information can enhance the medical tourism industry in the province and attract foreign patients since it can prevent the medical tourists from being confused and having a negative experience, it is recommended that IPDs include this section on their website.

### Strengths, limitations, and recommendations

As a strength point of this study, we used the census method and reviewed the websites of all hospitals of provinces with a high or low share of the medical tourism industry, which is more reliable than when sampling is used. Also, to the best of our knowledge, it is the first research in Iran, to investigate the association of websites’ quality of hospitals to their share of national medical tourism and ownership type of the hospitals as well. Therefore, further studies should be conducted to evaluate the quality of different aspects of medical tourism marketing in Iran. In addition, due to filtering matters, we could not access most of the websites of other countries, so we had to limit our study only to Iran. So, it is recommended to consider other websites of hospitals in other countries in future research and provide a more comprehensive and comparative vision to the global readers. Also, in addition to the hospital website quality, there are other factors affecting the share of the medical tourism industry in provinces which can be studied in future studies.

## Conclusion

Based on the findings of this study and the possible role of website quality in increasing provinces’ medical tourism development, the hospitals’ IPDs should design multilingual websites that can provide necessary information for prospective medical tourists and update them regularly, which may be very beneficial for the development of this industry in Iran more than before.

## Data Availability

All information is available whenever needed.
